# Sugar Estimation as an Alternative and Rapid Approach to Predicting Chemical Oxygen Demand (COD) in Produce Wash Water

**DOI:** 10.1111/1750-3841.70451

**Published:** 2025-08-06

**Authors:** Kevin C. Tarwa, Maria Moreno, Rohan V. Tikekar

**Affiliations:** ^1^ Department of Nutrition and Food Science University of Maryland College Park Maryland USA; ^2^ Del Monte Fresh Produce Co Jessup Maryland USA

**Keywords:** chemical oxygen demand, produce safety, produce washing, sanitizers

## Abstract

**ABSTRACT:**

Monitoring chemical oxygen demand (COD) during washing of fresh produce is critical to maintaining the antimicrobial efficacy of sanitizers. However, traditional COD analysis is time‐consuming (2–3 h) and costly. Since sugars released from fresh produce contribute to the overall COD, we investigated whether sugar estimation using the colorimetric sulfuric acid‐UV spectrometry method (SA‐UV method) can be a rapid, low‐cost, and field‐deployable method to predict the COD. The method relies on measurement of furfurals (absorbance at 315 nm, A_315_) formed by sulfuric acid‐induced oxidation of sugars (∼5 min). The correlation between A_315_ and measured COD was assessed using the coefficient of determination (*R* ^2^) for a linear regression model. A linear prediction model was subsequently developed. We first demonstrated this method with glucose, fructose, and sucrose (0–100 mg/L) solutions. Actual produce (grape, cantaloupe, and pineapple) wash water samples (> 15 samples per commodity) from a facility were then tested to validate this method. Strong positive associations were observed between A_350_ versus measured COD in all three sugar solutions (*R* ^2 ^> 0.91) as well as for grape (*R* ^2^ = 0.82) and cantaloupe (*R* ^2^ = 0.85) wash samples; while a relatively weaker correlation was observed for pineapple (*R* ^2^ = 0.62). Similar trends were observed between the measured and predicted COD of all three sugar solutions (*R* ^2 ^> 0.95) and produce commodities (grape: *R* ^2^ = 0.82; cantaloupe: *R* ^2^ = 0.87; pineapple: *R* ^2^ = 0.65). Compared to turbidity measurements (*R* ^2^ range 0.17–0.59), this method was more accurate (*R* ^2^ range 0.65–0.82) as a predictor of COD.

**Practical Applications:**

It is critical to monitor chemical oxygen demand (COD) levels in fresh produce wash water because elevated levels are associated with decreased antimicrobial efficacy of applied sanitizing agents. However, traditional COD measurements are time‐consuming (2–3 h), which creates a burden for wash operations, especially under extreme wash water conditions (high COD). The results from this study offer an alternative approach for estimating the COD present in wash water and can be useful for produce wash operations because of the minimal time required, low cost of reagents and supplies, and minimal exposure to toxic chemicals.

## 1 Introduction

Fresh produce is becoming increasingly recognized as a source of foodborne outbreaks since thermal processing as a complete “kill step” is generally not common in fresh produce production due to quality deterioration. Some of the major causes of foodborne outbreaks related to fresh produce are caused by contamination with human pathogens, such as Norovirus, *Escherichia coli* O157:H7, *Listeria monocytogenes*, and *Salmonella* (Lin et al. 1996; Gurtler and Gibson 2022). The burden created by foodborne outbreaks is attributed to economic loss, increased healthcare costs, loss of productivity, reductions in the quality of life, and increased mortality (López‐Gálvez et al. 2021).

Post‐harvest washing is critical to remove any residual soil, leaf debris, and foreign materials remaining on the surface of fresh produce. Washing produce with potable water can help remove microorganisms, but only to a certain degree. Therefore, sanitizing agents are generally applied to the wash water to prevent water‐mediated cross‐contamination between clean and contaminated produce. To better estimate the efficacy of the sanitizing agent used in wash water, it is critical to understand the sanitizer's disinfection kinetics, which are based on the disinfectant dose and contact time (Banach et al. 2015). Due to their low cost and sanitation efficiency in food processing, chlorine‐based disinfectants are often used as an antimicrobial agent in fresh produce wash water. However, the effectiveness of chlorine is influenced by various parameters such as pH, temperature, produce commodity, and fresh produce microflora (Chinchkar et al. 2022). One of the major concerns is the highly reactive nature of chlorine with organic matter, which can generate hazardous disinfection by‐products (DBPs), such as trihalomethanes (Petri et al. 2021).

Chemical oxygen demand (COD) is a measure of all forms of organic matter present (biodegradable and non‐biodegradable) (Kadhum et al. 2021). Organic load present in produce wash water can be attributed to residual soil, leaf debris, and tissue exudates resulting from external damage (Su et al. 2022). Monitoring the levels of COD present during produce washing is critical to maintaining the antimicrobial efficacy of chlorine. However, traditional COD measurements are time‐consuming (∼2–3 h), which can create a burden for produce wash operations (Geerdink et al. 2017). Elevated COD levels present during produce washing are shown to minimize the sanitizing efficacy of chlorine‐based sanitizers due to the rapid depletion of free chlorine (FC) available to inactivate potential microorganisms. Teng et al. (2018) investigated the sanitizing efficacy of chlorine to inactivate *E. coli* O157:H7 under stabilized washing conditions. The results indicated that the sanitizing efficacy of chlorine was influenced by the organic load present, and a minimum of 0.5 and 7.5 mg/L FC was required to achieve a 5‐log reduction at 0 and 900 mg/L COD, respectively. Another study evaluated the inactivation of *Salmonella, E. coli* O157:H7, and non‐O157:H7 Shiga toxin‐producing *E. coli* dependent on FC concentration, contact time, and organic load (Shen et al. 2013). Lettuce extract (LE) or tomato extract (TE) was prepared and applied to chlorine solutions as organic simulators. The results indicated that increasing the LE from 0 to 1.0% in the wash caused the FC to instantaneously decrease from 8.0 to 0.09 mg/L. With increasing organic load from 0%–0.2% LE or TE, there was also an increase in initial FC concentrations and contact time required to inactivate the pathogens because of the high reactivity of chlorine with organic matter.

Fresh produce is abundant in sugars such as glucose and fructose that can potentially increase organic load if leached out into the wash water system, resulting from external damage. Therefore, we hypothesized that sugar estimation can be a reasonable predictor of the overall COD present in the wash water. There are several rapid colorimetric methods available to measure sugar concentrations in water, such as the Anthrone method (Dreywood 1946) and periodic acid‐schiff stain method (Schiff 1866). However, one of the most reliable, rapid, and well‐known colorimetric methods is the phenol‐sulfuric acid method (Le and Stuckey 2016). This method relies on dissolving the sugar and phenol in water, followed by the addition of concentrated sulfuric acid (H_2_SO_4_). The heat generated from the reaction promotes the dehydration and formation of furfural derivatives, resulting in the formation of orange‐yellow complexes (Dubois et al. 1956). Nevertheless, this method is not readily adaptable in a packinghouses or processing facility where highly toxic chemicals such as phenols may not be desirable.

The sulfuric acid‐UV method (SA‐UV method) was developed as an alternative to the phenol‐sulfuric acid colorimetric method (Albalasmeh et al. 2013). Their rationale was to alleviate the health concerns posed by handling phenol, improve the accuracy of the measurements, enhance reaction time, and allow a direct correlation of light absorbance to total carbon concentrations in aqueous solutions (Albalasmeh et al. 2013). Their work was based on the work of Itagaki (1994), who determined that the aqueous solution of furfural has a UV‐light absorption maxima at 277 nm. The bathochromic shift in absorption maxima from 277 to 323 nm was caused by the presence of sulfuric acid in reaction with glucose and cellulose (Itagaki 1994).

Therefore, the aim of this work was to develop a prediction model to estimate the COD based on the absorbance measured using this SA‐UV method of aqueous solutions of various sugar standards (glucose, fructose, sucrose) and wash water samples from various commodities (grapes, pineapple, cantaloupe). Validation of the prediction model was assessed in terms of linearity, accuracy, and precision. Lastly, absorbance using the SA‐UV method to predict COD was compared to turbidity, another factor that is widely used in the fresh produce wash industry to indicate the need to add fresh water into the wash system.

## 2 Materials and Methods

### 2.1 Sugar Standard Solutions

Standard curves were made from D‐(+)‐glucose, anhydrous, 99% (Thermo Scientific, Waltham, MA, USA), D‐fructose (Spectrum Chemical, Gardena, CA, USA), and sucrose (Fisher Chemical, Fair Lawn, NJ, USA). Briefly, a 100 mg/L stock solution of each sugar was prepared by dissolving 0.01 g of the respective sugar in 100 mL of deionized water (DI water). The stock solution of each sugar was further diluted with DI water to reach a concentration range of 2–100 mg/L.

### 2.2 Produce Wash Samples

Produce wash samples were collected on separate occasions (every two weeks) and provided by Del Monte Fresh Produce Co. (Jessup, MD, USA) over eight months (January–August) of production. For the washing process, each commodity (grape, cantaloupe, and pineapple) was placed in micro‐bins filled with potable water containing sanitizer solution (commercially available sodium hypochlorite). Each commodity was washed at the indicated concentration of FC according to the facility's hazard analysis critical control plan (HACCP). Wash samples for each commodity were collected from the micro‐bin following the first, second, and third use, respectively.

### 2.3 Sulfuric Acid‐UV Method

The SA‐UV method was carried out based on the method developed by Albalasmeh et al. (2013) with slight modifications. Prior to the SA‐UV method, sugar standard solutions were vortexed for 10 s. Concentrated sulfuric acid (VWR International, Radnor, PA, USA) was then mixed with an aliquot of sugar standard solution at a ratio of 3:1 in a glass test tube for 2 min, followed by cooling to room temperature in an ice bath for 2 min. After that, UV light absorption was read at 315 nm using a UV‐spectrophotometer (SpectraMax M5^e^, Molecular Devices, LLC, San Jose, CA, USA). The same procedure was carried out for the produce wash samples, and all reference standards were prepared following the same procedure, except that the sugar aliquot was replaced with DI water.

### 2.4 Chemical Oxygen Demand

COD measurement was carried out in triplicate by the standard operating procedures (SOPs) developed by the Department of Environmental Science & Technology at the University of Maryland (College Park, MD, USA). Briefly, a 2 mL aliquot of sugar standard solution was added to Mercury‐free COD2 Digestion vials (Hach Company, Loveland, CO, USA) and inverted thrice. The COD vials containing the aliquot of sugar standard solution were then placed in a COD reactor (DRB200 Digital Reactor Block) heated at 150°C for 2 h. Following the digestion process, vials were placed in a dark environment and cooled to room temperature. Lastly, the colorimetric measure of COD concentration was read with the HACH DR 5000 spectrophotometer (Hach Company, Loveland, CO, USA) using the “435 COD HR 1500 mg/L” program stored on the equipment. Produce wash samples were prepared using the same procedure, and reference solutions were replaced with DI water.

### 2.5 Prediction of Chemical Oxygen Demand

A linear regression model using the least squares method was used to define the coefficient of determination (*R* ^2^). Measured COD was plotted as a function of absorbance (A_315_) for the sugar standards (glucose, fructose, and sucrose) and produce wash samples (grape, cantaloupe, and pineapple). This model was used to determine their correlation, in which a prediction model for COD was developed based on the slope‐intercept of the regression line (y=mx+b).

### 2.6 Turbidity Measurement

The turbidity of the produce wash samples was measured using a handheld device (Hanna Instruments, Woonsocket, RI, USA). The recorded turbidity values were then plotted as a function of measured COD to determine the correlation between turbidity and COD.

### 2.7 Method Validation

The validation of the SA‐UV method to predict COD was based on the method described by Albalasmeh et al. (2013). The following standards were used for the validation process: Linearity, precision, and accuracy. The linearity of the SA‐UV method is expressed as the coefficient of determination (*R* ^2^), indicating the proportion of the dependent variable (measured COD) that can be predicted from the independent variable (A_315_). The accuracy to predict COD based on the regression equation was assessed in terms of percentage recovery (*r*). *r* was determined as:

(1)

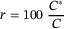

where [C*] represents the predicted COD determined by the regression equation for each respective carbohydrate and produce wash sample and [*C*] is their respective measured COD.

The precision of the SA‐UV method to predict COD is expressed in terms of the standard deviation (SD) within a series of triplicate measurements.

### 2.8 Statistical Analysis

The data reported in this study represent the mean and standard deviation of three independent replicates. Microsoft Excel (Microsoft Corporation, Redmond, WA, USA) was used for statistical analysis and storing all data points. Regression equations and the coefficient of determination (*R* ^2^) were quantified using Microsoft Excel. RStudio version 2024.09.1 (Boston, MA, USA) was used for one‐way analysis of variance (ANOVA) and Tukey post‐hoc testing to determine differences in the means of multiple groups at a significance level of 0.05. Before statistical analysis, wash samples with an A_315_> 2.4 were further diluted prior to re‐measuring their absorbance and COD because of the sensitivity of the method. Finally, the predicted COD for any wash samples with an A_315_> 2.4 identified were calculated based on their dilution factor.

## 3 Results

### 3.1 Measurement and Prediction of COD in Sugar Standards

**FIGURE 1:**
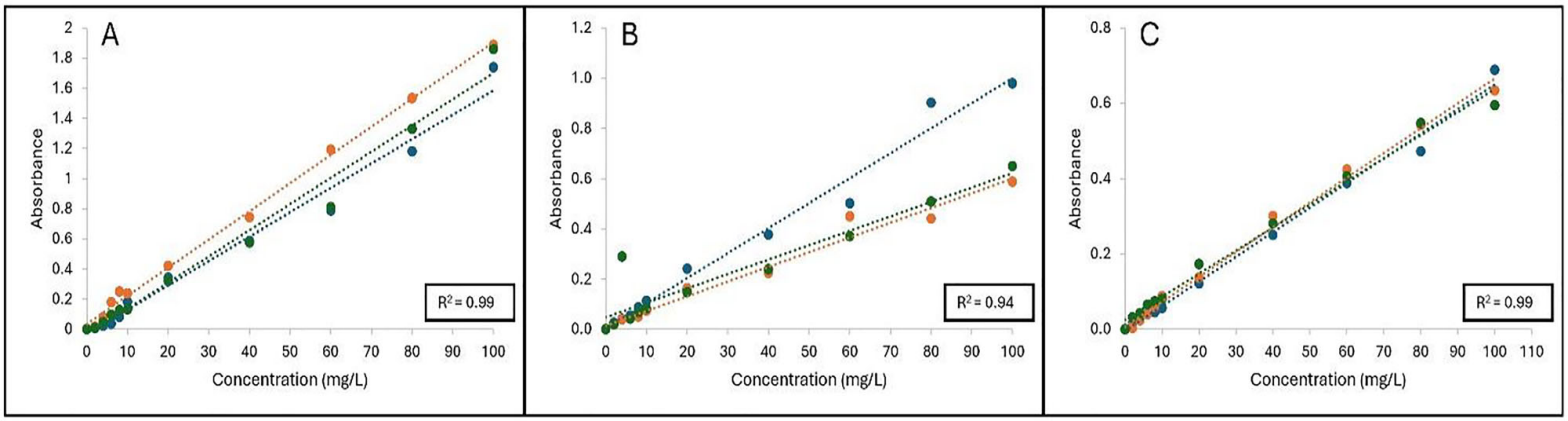
**A–C**:Linear regression curves plotted as absorbance as a function of concentration (mg/L) for (A) glucose, (B) fructose, and (C) sucrose, respectively. Each linear regression curve is represented as one independent replicate (*n* = 3) which is denoted as: Blue = R1, orange = R2, and green = R3. Coefficient of determination (*R* ^2^) is shown as the average of the three independent replicates.

Glucose, fructose, and sucrose were chosen as the sugar standards in this study because of their natural abundance in fresh produce (Lee et al. 1970), which could account for the increased organic matter released into produce wash systems resulting from tissue damage. Figure 1A–C indicates the linear regression curves obtained when plotting absorbance (A_315_) as a function of concentration (mg/L) for glucose, fructose, and sucrose, respectively. The coefficient of determination (*R* ^2^) indicated a strong linear correlation between the concentrations of the sugars and A_315_, in which the *R* ^2^ values for glucose, fructose, and sucrose were *R* ^2^ = 0.99, *R* ^2^ = 0.94, and *R* ^2^ = 0.99, respectively. The slopes and standard deviations obtained for glucose, fructose, and sucrose were 0.02 ± 0.00, 0.01 ± 0.00, and 0.01 ± 0.00, respectively. The slope obtained for glucose was significantly different than the slopes of fructose and sucrose (*p* < 0.05), while no significant differences were observed between the slopes of fructose and sucrose (*p* > 0.05). This contrasts with the results of Albalasmeh et al. (2013), where no statistical differences in the slopes of glucose, fructose, and sucrose were observed. It is worth noting that the wavelength used in this study for measured absorbance was at 315 nm, similar to the maximum absorption determined in the full UV light spectrum reported by Albalasmeh et al. (2013). Slight bathochromic shift differences can be observed depending on the concentration of sulfuric acid used. However, in their study, absorption maxima for all carbohydrate standards were seen at 315 nm, except for polygalacturonic acid (PGA) (297 nm).

**FIGURE 2:**
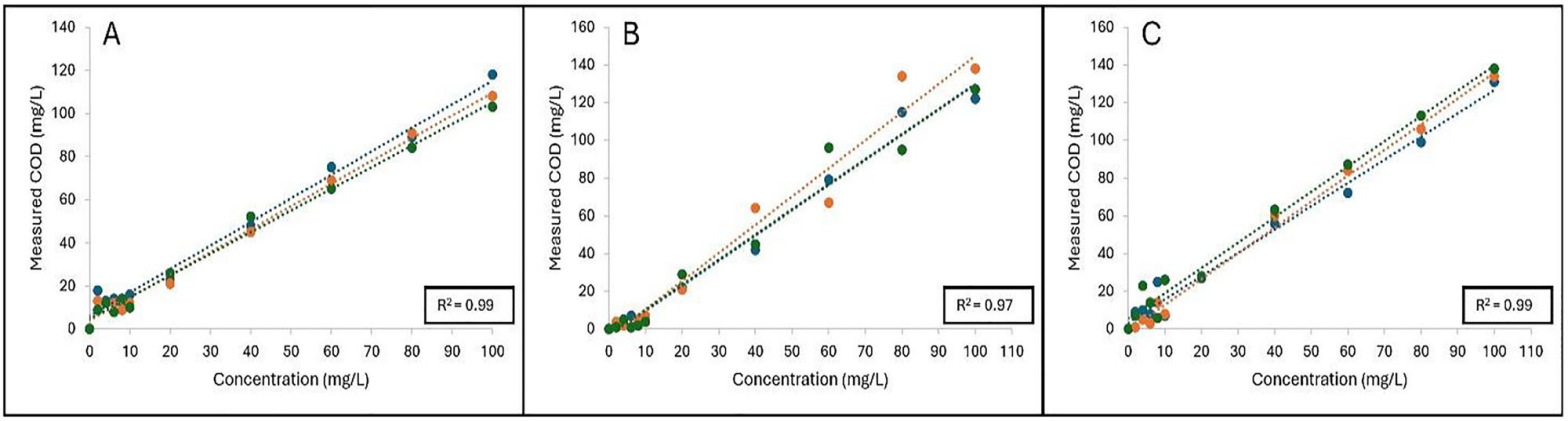
**A–C**: Linear regression curves plotted as measured COD (mg/L) as a function of concentration (mg/L) for (A) glucose; (B) fructose; and (C) sucrose standards, respectively. Each linear regression curve is represented as one independent replicate (*n* = 3) which is denoted as: Blue = R1, orange = R2, and green = R3. Coefficient of determination (*R* ^2^) is shown as the average of the three independent replicates.

Figure 2A–C indicates the linear regression curves obtained when plotting measured COD (mg/L) as a function of concentration for glucose, fructose, and sucrose, respectively. Concentration was also positively associated with the measured COD of the sugar standards, in which the *R* ^2^ values for glucose, fructose, and sucrose were *R* ^2^ = 0.99, *R* ^2^ = 0.97, and *R* ^2^ = 0.99, respectively. The slopes obtained for glucose, fructose and sucrose were 1.05 ± 0.04, 1.38 ± 0.09, and 1.31 ± 0.07, respectively. Significant differences were observed when comparing the slope of glucose to fructose and sucrose (*p* < 0.05), while no significant differences in the slopes were observed between fructose and sucrose (*p* > 0.05).

Measured COD was then plotted as a function of A_315_ to develop a linear regression model to predict COD based on A_315_ measured using the SA‐UV method. The following regression equations developed for the respective carbohydrate standards were:

(2) Glucose:y=62.28x+5.57

(3) Fructose:y=192.87x−5.85

(4) Sucrose:y=203.60x+0.99

in which *y* is the predicted COD, and *x* is the A_315_ measured.

**FIGURE 3:**
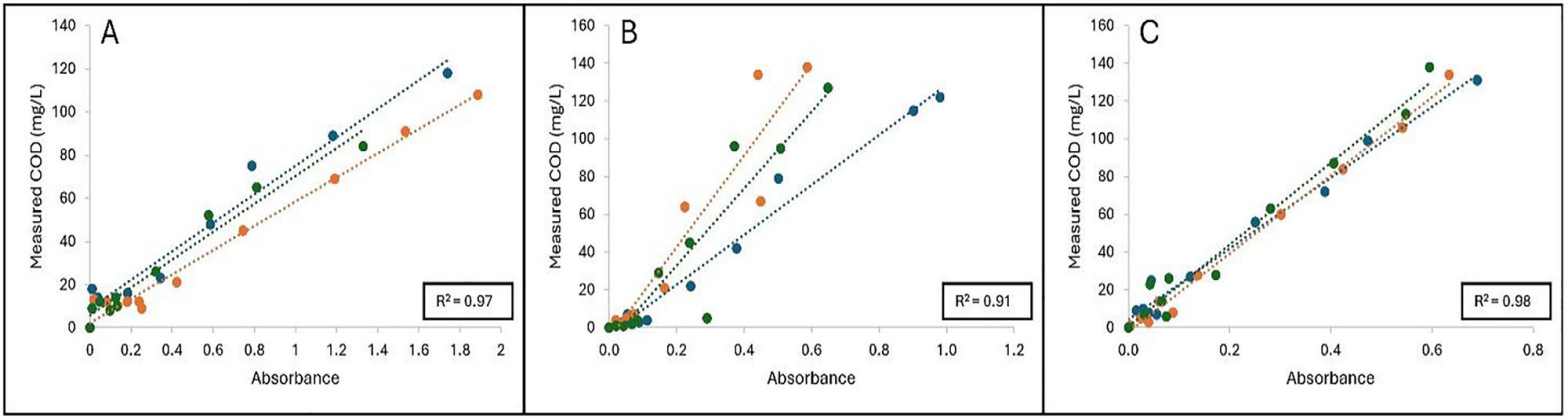
**A–C**: Linear regression curves plotted as measured COD (mg/L) as a function of absorbance for (A) glucose, (B) fructose, and (C) sucrose standards, respectively. Each linear regression curve is represented as one independent replicate (*n* = 3) which is denoted as: Blue = R1, orange = R2, and green = R3. Coefficient of determination (*R* ^2^) is shown as the average of the three independent replicates.

Figure 3A–C indicates the linear regression curves obtained when plotting the measured COD as a function of A_315_ for glucose, fructose, and sucrose. Strong positive linear correlation between measured COD and A_315_ was observed for glucose (*R* ^2^ = 0.97), fructose (*R* ^2^ = 0.91), and sucrose (*R* ^2^ = 0.98). However, differences in sensitivity of the method can be observed when comparing the slopes of the sugar standards. The slope for measured COD versus A_315_ obtained for glucose (62.28 ± 5.34) was significantly lower (*p* < 0.05) compared to the slopes for fructose (192.87 ± 56.02) and sucrose (203.60 ± 14.65). However, no significant differences between the slopes of fructose and sucrose were observed (*p* > 0.05). The smaller slope of glucose observed in this study indicates a lower sensitivity towards the method in comparison to a higher sensitivity observed for fructose and sucrose. For glucose, smaller changes in the measured COD were detected as the sample A_315_ increased. However, for sucrose and fructose, larger increases in their measured COD values were observed with an increase in their respective A_315_ values.

**FIGURE 4:**
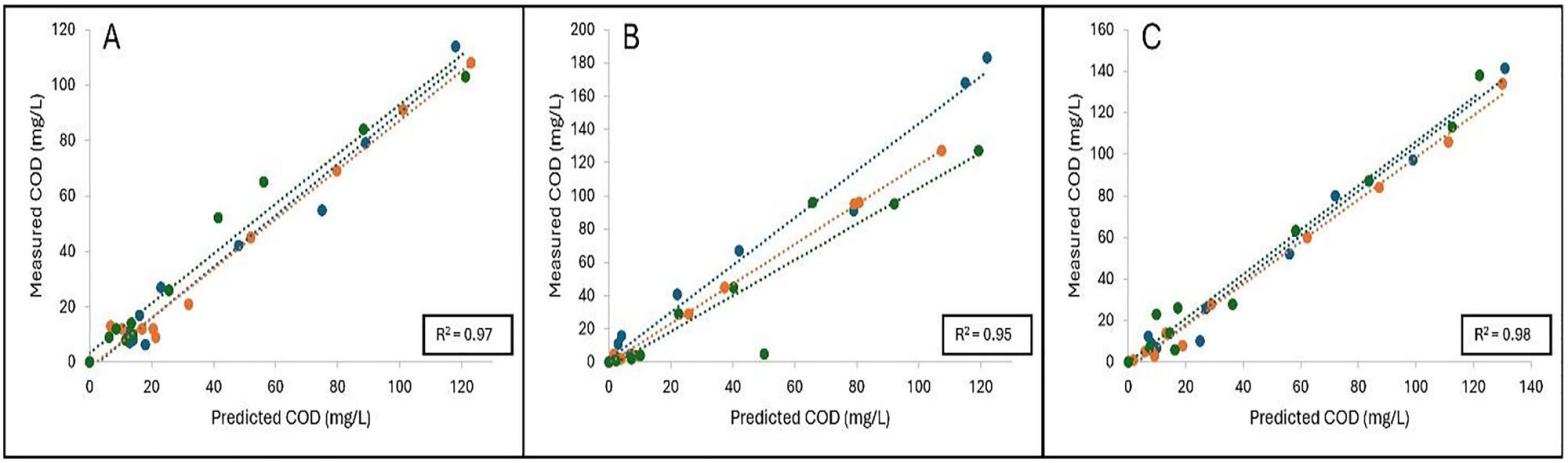
**A–C**: Linear regression curves plotted as measured COD (mg/L) as a function of predicted COD (mg/L) for (A) Glucose, (B) Fructose, and (C) Sucrose standards, respectively. Each linear regression curve represents one independent replicate (*n* = 3), and coefficient of determination (*R* ^2^) is shown as the average of three independent replicates.

Figure 4A–C represents the linear regression curves plotted as measured COD as a function of predicted COD based on the regression equations obtained for glucose, fructose, and sucrose standards, respectively. Slopes obtained for glucose, fructose, and sucrose were 0.90 ± 0.02, 1.23 ± 0.18, and 1.05 ± 0.03, respectively. Significant differences were observed when comparing the slope of glucose to sucrose and fructose (*p* < 0.05), while no significant differences were observed for sucrose and fructose (*p* > 0.05). However, strong positive linear correlations were observed between the measured COD and predicted COD values of glucose (*R* ^2^ = 0.97), fructose (*R* ^2^ = 0.95), and sucrose standards (*R* ^2^ = 0.98), verifying the potential of this method to predict COD.

### 3.2 Measurement and Prediction of COD in Produce Wash Samples

**FIGURE 5:**
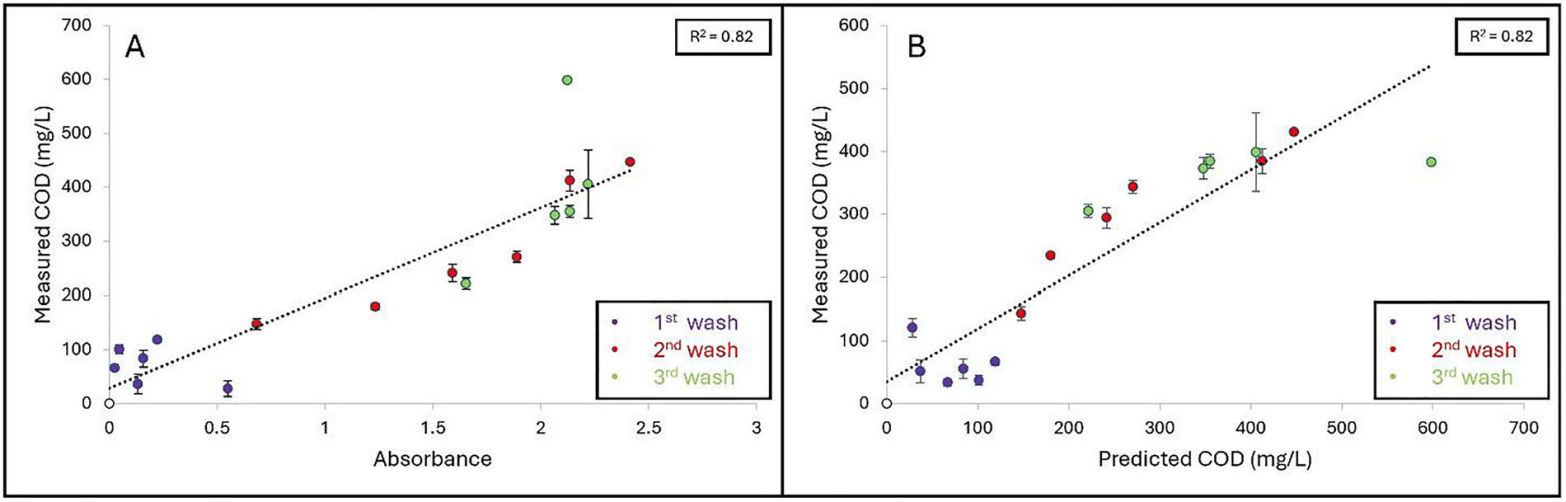
**A–B**: Linear regression curves plotted as (A) Absorbance as a function of measured COD (mg/L) and (B) Measured COD (mg/L) as a function of predicted COD (mg/L) based on wash samples collected from various grape washing water that is reused for multiple cycles (First, second, and third). Coefficient of determination (*R* ^2^) is shown as the average of three independent replicates.

**FIGURE 6:**
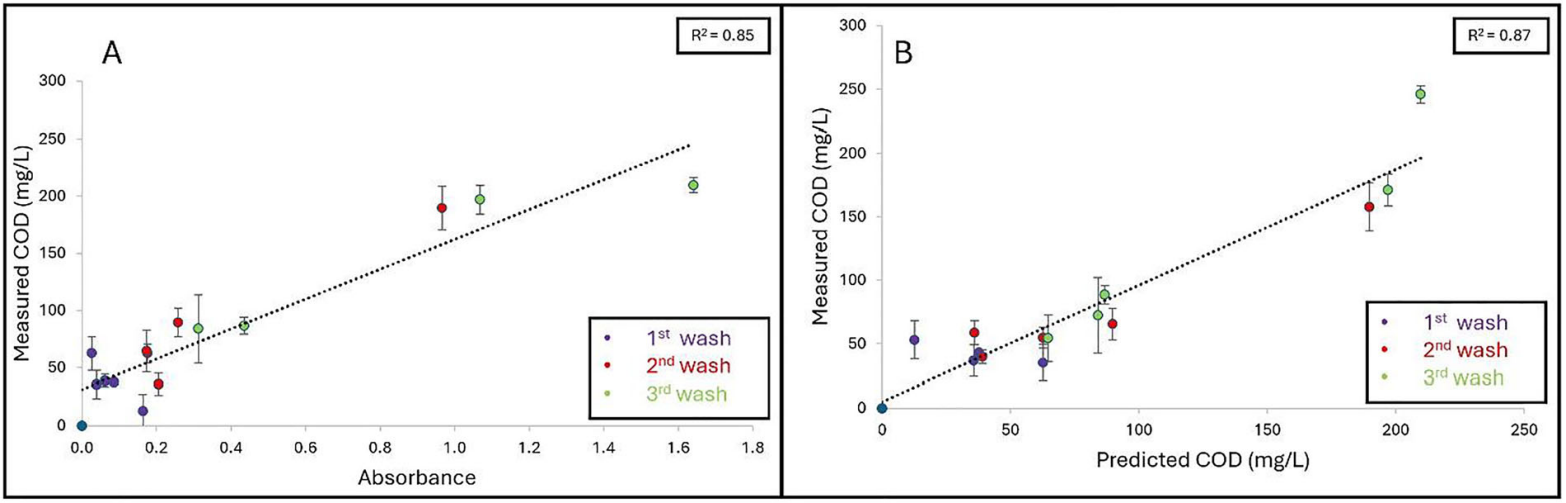
**A–B**: Linear regression curves plotted as (A) Absorbance as a function of measured COD (mg/L) and (B) Measured COD (mg/L) as a function of predicted COD (mg/L) based on wash samples collected from cantaloupe washing water that is reused for multiple cycles (First, second, and third). Coefficient of determination (*R* ^2^) is shown as the average of three independent replicates.

**FIGURE 7:**
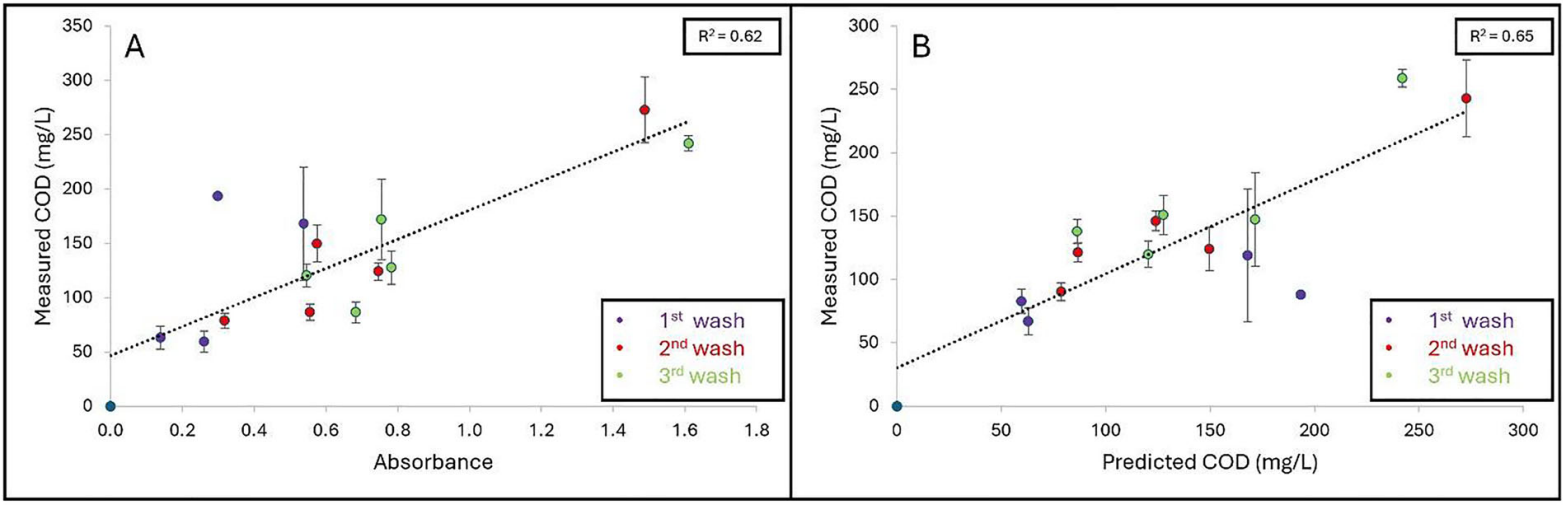
**A–B**: Linear regression curves plotted as (A) Absorbance as a function of measured COD (mg/L) and (B) Measured COD (mg/L) as a function of predicted COD (mg/L) based on wash samples collected from various pineapple washing water that is reused for multiple cycles (First, second, and third). Coefficient of determination (*R* ^2^) is shown as the average of three independent replicates.

Grape, cantaloupe, and pineapple wash samples were collected on six separate occasions over a span of 8 months (January–August) from 3 different washing stages (1st, 2nd, and 3rd) to represent how organic load can increase throughout multiple cycles of water usage. Figures 5, 6, and 7A represent the linear regression curves plotted as measured COD (mg/L) as a function of A_315_ for grape, cantaloupe, and pineapple wash samples, respectively. Strong positive linear correlations between measured COD and A_315_ were observed for grape (*R* ^2^ = 0.82) and cantaloupe (*R* ^2^ = 0.85) wash samples, while a weaker correlation was observed for pineapple wash samples (*R* ^2^ = 0.62). High variation was observed in A_315_, and the measured COD of the produce wash samples can be attributed to dissimilarities in the abundance of sugars in the produce commodities. In addition, the amount of tissue damage could also contribute to the organic load present in the wash water.

Regression equations were then developed from the linear curves obtained from measured COD as a function of A_315_ for the respective produce wash samples (Figures 5, 6, and 7A) to determine whether this method could be applicable to an industrial wash setting. The following regression equations obtained were:

(5) Grape:y=166.22x+29.64

(6) Cantaloupe:y=130.54x+31.68

(7) Pineapple:y=130.36x+48.82

in which *y* is the predicted COD and *x* being the A_315_ value. Significant differences (*p* < 0.05) were observed between the slopes of all three produce commodities: Grape (166.22 ± 1.64), cantaloupe (130.54 ± 9.96), and pineapple (130.36 ± 15.20). For wash samples with an absorbance > 2.4, dilutions were required because of the sensitivity of the method outside of this range. Strong positive correlations were observed between the measured COD and predicted COD values for the grape (*R* ^2^ = 0.82) (Figure 4B) and cantaloupe (*R* ^2^ = 0.87) (Figure 5B). A weaker correlation between measured COD versus predicted COD was observed for pineapple wash samples (*R* ^2^ = 0.65) (Figure 6B). The SA‐UV method's ability to predict COD is dependent on the amounts of sugar released during produce washing. In general, pineapple surfaces are less prone to damage compared to grapes and cantaloupes. Therefore, we suspect that the weaker correlation observed for the pineapple wash samples was attributed to lower amounts of sugar being released into the wash solution. These results highlight the importance of developing respective regression equations to predict COD based on the produce commodity washed.

### 3.3 Method Validation

The validation of the SA‐UV method to predict COD was evaluated based on linearity, accuracy, and precision. The linearity of the method is represented by the coefficient of determination (*R* ^2^) obtained when plotting measured COD (mg/L) as a function of A_315_. The accuracy of the SA‐UV method to predict COD based on the regression equation was assessed in terms of percentage recovery (*r*) (Equation 1), and values within close to 100% were considered accurate. Precision of the method is assessed in terms of the standard deviation, in which a generally low‐standard deviation indicates high precision. The accuracy and precision of the SA‐UV method to predict COD are shown in Table 1. Overall, the accuracy of the method was not systematic, and fluctuations in the accuracy can be seen throughout the concentration range used in this study (2–100 mg/L). Table 2 represents the accuracy and precision of the method to predict COD for produce wash samples based on their respective washing stage. High accuracy was observed for all produce wash samples, which was represented by the percent recovery (*r*) for their respective washing stages being near 100%. Higher standard deviations (low precision) can be attributed to various factors, such as dissimilarities in the amount of ripeness of the produce through the sampling period, affecting sugar content. Also, differences in the amount of tissue damage on the surface of the produce can contribute to high organic load in the wash water. Therefore, it is important that in an industrial application, produce wash operators identify the acceptable range of COD in the wash water based on the washing stage to ensure that the FC available is effectively reducing the microbial load on the produce surface.

**TABLE 1:** Accuracy and precision of SA‐UV method to predict the COD of carbohydrate standards. Accuracy is represented as the percent recovery % (*r*) for the average of three independent replicates while precision is represented as the standard deviation (SD) of the predicted COD values of three independent replicates.

	Glucose		Fructose		Sucrose	
Concentration (mg/L	SD	Percent recovery (*r, %)*	SD	Percent recovery (*r, %*)	SD	Percent recovery (*r, %*)
**2**	0	46.2	1	−100.0	3	83.3
**4**	2	75.0	28	600.0	2	61.5
**6**	4	109.1	1	100.0	3	137.5
**8**	5	125.0	4	233.3	3	86.7
**10**	3	130.8	4	240.0	3	114.3
**20**	3	121.7	10	125.0	5	107.1
**40**	6	93.8	16	96.0	5	96.7
**60**	14	90.0	13	97.5	4	103.7
**80**	11	102.3	48	98.3	8	100.9
**100**	5	108.2	41	106.2	10	97.8

**TABLE 2:** Accuracy and precision of SA‐UV method to predict the COD of produce wash samples corresponding to their respective washing stages. Accuracy is represented as the percent recovery % (*r*) for the average of three independent replicates while precision is represented as the standard deviation (SD) of the predicted COD values of three independent replicates.

	Grape		Cantaloupe		Pineapple	
Washing stage	SD	Percent recovery % (*r*)	SD	Percent recovery % (*r*)	SD	Percent recovery % (*r*)
**1st**	32	82.2	5	121.1	21	83.5
**2nd**	105	107.8	32	81.0	57	108.4
**3rd**	37	95.6	55	79.8	53	115.3

### 3.4 Turbidity Measurements

**FIGURE 8:**
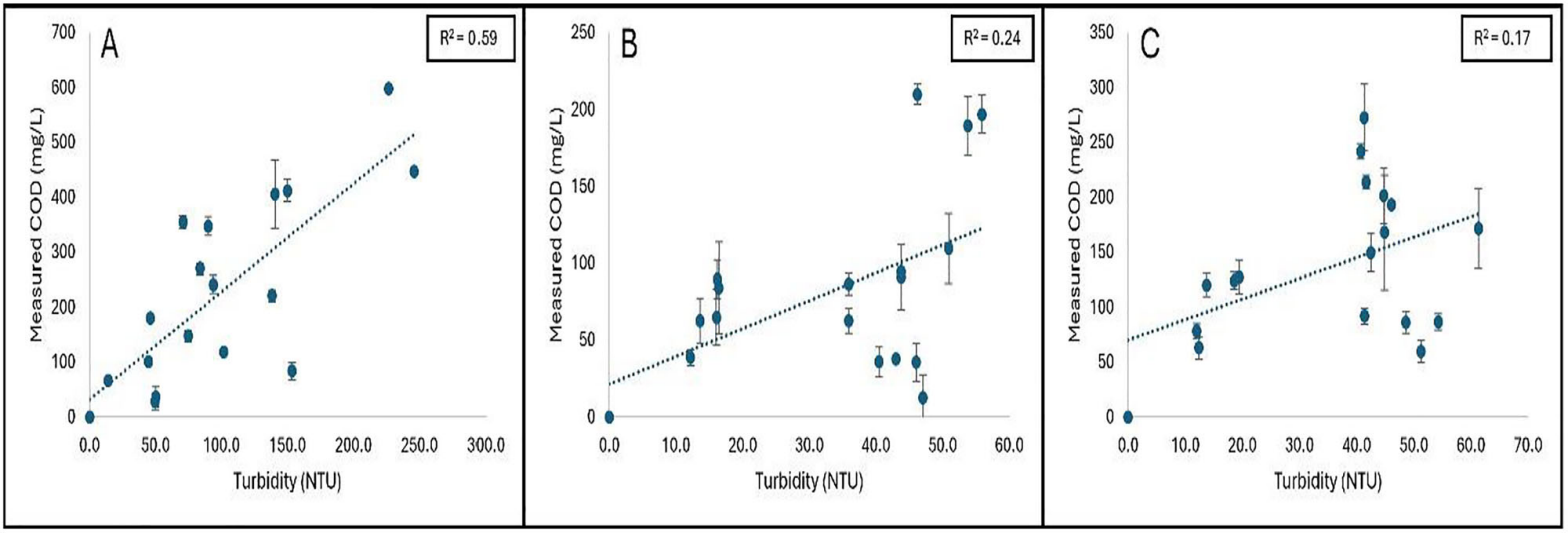
**A–C**: Linear regression curves plotted as measured COD (mg/L) as a function of turbidity based on wash samples collected from (A) Grape washing line, (B) Cantaloupe washing line, and (C) Pineapple washing line. Coefficient of determination (*R* ^2^) is shown as the average of three independent replicates.

Turbidity is a rapid, and easy‐to‐obtain measurement of the intensity of light scattered by particulates in an aqueous solution (Li et al. 2019). In general, the turbidity of a produce wash line can increase due to excess residual soil on the surface of the targeted produce commodity, causing operators to replenish the wash water. Therefore, in this study, our method of using absorbance to predict COD was compared to a more common method of turbidity to predict COD, since it is commonly used as an indicator to replenish the wash water. In this study, the turbidity of the collected wash samples was measured, and linear regression curves were plotted as measured COD (mg/L) as a function of turbidity (NTU), and the coefficient of determination (*R* ^2^) was used to assess their correlation. Figure 8A–C represents the linear regression curves plotted as measured COD as a function of turbidity for grape, cantaloupe, and pineapple wash samples, respectively. Overall, a weak linear correlation was observed between the turbidity of the wash samples with respect to their measured COD values: Grape (*R* ^2^ = 0.59), cantaloupe (*R* ^2^ = 0.24), and pineapple (*R* ^2^ = 0.17). This result may highlight the limitation of replenishing wash water based on its appearance (turbidity) and could alleviate the drastic water consumption used in the produce wash industry.

## 4 Discussion

The results of this study demonstrate a viable alternative approach to predicting COD in produce wash water based on sugar estimation obtained using the SA‐UV method. One of the main benefits of using this method to predict COD is the lower time required to perform the assay, in which traditional COD measurements required ∼2–3 h while the SA‐UV method requires <5 min to perform. In comparison to traditional COD measurements, this method is lower cost, only requiring minimal amounts of reagents (<3 mL sulfuric acid per assay) and a spectrophotometer. Traditional COD measurements require the use of specific COD vials, which are costly, and smaller washing facilities may have to rely on a second‐party laboratory to perform the tests. Also, this SA‐UV method can be adaptable to a produce wash facility because of the low health risks to workers requiring minimal personal protective equipment (PPE) such as gloves and proper eye protection.

It is worth noting that the SA‐UV method is not a direct measure of the disinfection capacity of wash systems. Monitoring the disinfection capacity of sanitizers applied during produce washing can be quantified using titration kits and oxidation–reduction potential (ORP). The SA‐UV method to predict COD using sugar estimation provides washing operations with an overall understanding of COD levels in their wash water. Since the disinfection potential of a sanitizer is affected by the COD of water, our method complements existing methods of measuring disinfection potential. Produce wash operations may develop SOPs based on various ranges of COD levels that maximize the efficacy of the sanitizing agent. The presence of high organic load during produce washing can accumulate DBPs due to the high reactivity of chlorine with organic matter. Therefore, having a rapid COD detection method employing the SA‐UV method allows processors to maintain the safety of their workers.

Positive linear correlations were observed between A_315_ and measured COD for the respective sugar standards and produce wash samples. These results are comparable to the results of Li et al. (2019), who observed a linear correlation between total soluble solids (TSS) and COD (*R* ^2^> 0.87). However, their study indicated a strong linear correlation between turbidity and COD (*R* ^2^ = 0.85) as well. In our study, weak observations in the correlation between the turbidity and COD values for the produce commodities were observed. Differences in our results may be attributed to the fact that their produce commodities (romaine lettuce, iceberg lettuce, and carrots) were cut prior to washing, which may have led to increased turbidity in the wash water. In our study, the produce commodities were cut following the washing stages, so the increase in turbidity could have only been attributed to residual soil on the produce or tissue exudates resulting from damage. Another study also utilized turbidity as an indicator for COD in treated effluent containing residual suspended microalgae, with results suggesting a strong correlation between the COD and the turbidity (*R* ^2^ = 0.97) (Nguyen et al. 2014). Therefore, future work in developing a linear regression curve using fixed turbidity measurements may be required to better understand the correlation between the two parameters (COD and turbidity).

One limitation of this method is that it does not quantify the exact amount of sugars present in the wash solution and only estimates the relative abundance of sugars. Although fruits and vegetables are abundant in sugars, the composition of sugars present varies among different commodities. This can explain the variation in the correlation observed between the measured COD versus predicted COD of the produce wash samples, which was *R* ^2^ = 0.65–0.85. Therefore, the SA‐UV method to predict COD during produce washing is not a one‐size‐fits‐all approach and must be commodity‐specific. The overall goal of this method is not to provide wash operations with an exact COD value but an approximate COD range in their wash systems. Therefore, having this understanding of the COD ranges in the washing systems can allow operations to develop SOPs that are specific to their wash processes. Another limitation of this method is that it depends on the reaction of concentrated sulfuric acid with the sugars present. However, sulfuric acid can react with other organic compounds present, such as organic acids, vitamins, etc. Therefore, the interaction between sulfuric acid and other organic compounds present needs to be further investigated.

## 5 Conclusion

Natural sugars can leach out from fresh produce during washing and contribute to increased organic load and COD of wash water, both of which can affect the sanitizer efficacy and increase microbial food safety risk. Thus, we investigated whether rapid estimation of sugar content using the SA‐UV method in wash water can be a rapid predictor of COD of wash water. Based on the linearity of response and a relatively strong coefficient of determination, we found that sugar estimation using this method is a reasonable predictor of COD levels during produce washing. However, we also found variations in the slopes of the regression equations generated for wash waters from grape, cantaloupe, and pineapple, as factors such as ripeness or surface characteristics of the produce can affect the release of sugar in the wash water. Therefore, this method needs to be calibrated for each specific commodity and washing process. A relatively weaker correlation between the turbidity of the wash samples and measured COD was observed compared to the sugar estimation method described here, indicating that the latter may be a predictor of COD. This method allows processors to effectively monitor COD levels present during produce washing, which can ensure the safety of their products while maintaining a safe work environment.

## Author Contributions

**Kevin C. Tarwa**: conceptualization, methodology, software, data curation, investigation, formal analysis, writing –review and editing, writing – original draft, visualization, validation. **Maria Moreno**: conceptualization, investigation. **Rohan V. Tikekar**: conceptualization, methodology, supervision, writing – review and editing, project administration, funding acquisition, investigation.

## Conflicts of Interest

The authors declare no conflicts of interest.
